# The New General Hospital, Toronto

**Published:** 1913-09-27

**Authors:** 


### THE NEW GENERAL HOSPITAL, TORONTO.

We have received some interesting details concerning the above hospital from Mr. Conrad W. Thies, Hon. Secretary British Hospitals Association, and have much pleasure in publishing the following summary :—

On the occasion of my last visit to Canada in 1909, I was shown over the site of the proposed new General Hospital in Toronto by Dr. Brown, who was at that time the superintendent of the old hospital, but has since removed to Detroit, where he is now superintending the erection of the new General Hospital in that city. It happened that my recent visit, a few days since, was on the day that the new hospital in Toronto was receiving its first instalment of fifty patients, and I observed the careful and expeditious manner in which these sick people were removed to their new quarters. Most of them were carried in the motor ambulances which will form part of the regular equipment of the new hospital. Dr. C. K. Clarke, the present superintendent, accompanied me over the hospital, and kindly furnished me with information respecting the history of the institution.

### Genesis of the New Buildings.

For many years past it had been realised by the managers that the old hospital was quite unable adequately to provide for the medical and surgical needs of the increasing population of Toronto and the country around. In 1904 Mr. J. W. Flavelle, who had been elected chairman of the Board of Trustees, at once made his influence felt, and gathered around him several of the most wealthy and influential citizens. At his suggestion a special report was prepared, which strongly recommended the erection of an entirely new hospital upon a fresh site, and pointed out that the demands of modern medical and surgical science could not possibly be properly met by any adaptations or additions to the existing hospital buildings. Some wealthy citizens at once promised contributions, and a new site of over nine acres adjoining the University was acquired at a cost of some $600,000 (£120,000). A scheme for the new hospital was then formulated, which involved an estimated expenditure of about $3,000,000 (£600,000).

The University of Toronto made a donation sufficient to cover the cost of the land, while grants from the Provincial Government and City Council, together with gifts by various wealthy citizens, promptly provided the necessary funds. Mention must here be made of Mr. Cawthra Mulock, who started the subscription list with a donation of $100,000 to defray the cost of the outpatient block. Then Mr. J. C. Eaton generously undertook to build and equip the surgical wing at a cost of about $300,000; while two ladies—the Misses Shields—provided the cost of the casualty and receiving departments, including the equipment and endowment of a complete ambulance service. The citizens were determined that Toronto should possess a hospital worthy of its reputation as "The Queen City" of the Dominion of Canada.

### A Comparison with New "King's."

The foundation-stone of the new hospital was laid by Earl Grey, the Governor-General, on April 11, 1911, and the work of building was energetically carried on. The formal opening took place on June 19 last. The entire cost of the new hospital and its equipment was raised before the opening ceremony, an achievement of which any city might well be proud. The hospital now provides accommodation for some 700 patients, 200 nurses, thirty resident medical officers, and 200 male and female servants, together with ample provision for a large number of medical students. It comprises eleven separate buildings, and in many respects it reminded me of the new King's College Hospital at Denmark Hill, which I had visited a few weeks previously. Provision is made for 450 medical and surgical beds; special departments for eye, throat, nose, and ear, 50 beds; and gynæcology, 50 beds; while 150 beds are provided for paying patients.

The main building, which contains the administrative department and medical and surgical wards, is three storeys high and 620 feet in length. The wards are well lighted and ventilated, while ample provision is made for heating, which is very essential in this country, where the variations of temperature are so great.

### Novelties of Equipment.

I was much impressed by several novel features of the equipment. For instance, every bed is provided with an electric call; upon a button being pressed by any patient a coloured electric light appears on an index board at the entrance to the ward, also in the service room and corridor. This light cannot be extinguished until the nurse goes to the patient's bed. I noticed many other useful and ingenious appliances, some of which might very well be employed in British hospitals. The floors of the corridors and wards are of concrete and are covered with thick red linoleum, similar to that used on German battleships. It is claimed for this kind of flooring that it is noiseless, durable, easily kept clean, and pleasing to the eye.

The walls of the wards, corridors, etc., are of cement coated with Paripan paint, tiled dados being provided for the staircases. The roof-gardens over the ward blocks are a pleasing feature, and, as elevators run up to these roofs and all the beds are fitted with large rubber-coated castors, the patients can be very easily moved to these roof-gardens.

The electric lighting of the wards and operating theatres is on the indirect principle, and thus a soft diffused light is obtained, and all glare is avoided.

### Sidelights on Special Departments.

On the surgical side each ward has its own operating-theatre. The surgical rooms, laboratories, etc., are models, and show a careful study of modern requirements. The equipment of bedsteads, lockers, screens, table, etc., are of metal and glass, no wood being employed. Ample accommodation is provided for the special departments. The provisions for radiography, pathology, hydrotherapeutics, Swedish exercises, sterilising rooms, etc., are on a most generous scale. The various buildings are all connected by covered ways. The nurses' home is approached by a beautiful reception-room, and the rooms generally are equal to what is provided in a high-class modern hotel—ample bathrooms on each floor, with a spacious and handsome dining hall for 200 nurses, library, recreation rooms, and lounge; a spacious garden, with tennis lawns, is also to be provided. Special accommodation is made for medical students and the visiting physicians and surgeons, also a spacious amphitheatre for clinics. The kitchens, store-rooms, laundry, and other offices are all on a corresponding scale of completeness. In the power-house are the steam and electrical equipments; the boilers and engines are of 2,000 h.p., and the electrical turbines develop ample current for all purposes: electricity is here employed in many ways for saving labour that are both novel and ingenious. Time will not permit me to refer in detail to the out-patient and emergency departments. The ambulance department connected therewith has three motor ambulances, which are available night and day.

The buildings are of brick of great hardness and fine finish, relieved with stone ornamentation. The style of architecture is early Georgian. At present the general effect is, I think, rather heavy and severe, but, doubtless, when the grounds are laid out with lawns, trees, shrubberies, and gardens, the appearance will be greatly improved.

### The New Pay Wards.

The provision which is made for 150 paying patients, as Sir William Osler so forcibly stated in his presidential address at the recent Conference of the British Hospitals Association at Oxford, is an essential part of every general hospital, both in Canada and the United States. This department is entirely separated from the rest of the hospital, and is complete in itself. It is contained in a five-storey building, which is furnished and equipped in the most perfect manner for the comfort of patients who can afford to pay for their treatment. It will be more than self-supporting, and, indeed, it is anticipated that it will prove a substantial source of income. I am assured that it has frequently happened that the well-to-do patients who have been treated in this department have proved generous contributors to the funds for the maintenance of the general work of the hospital.

### "Words of Criticism."

Before concluding these remarks, I must venture to add a word or two of criticism. In the first place, as regards the site, I am strongly of opinion that a better position for the new hospital might have been found a little further distant from what will probably in a few years be the centre of the city; for instance, a little to the north of the University there is gently undulating land, which would have been far less costly than the present site, which necessitated the removal of some 200 houses. The front entrance is not sufficiently imposing, and the doorway is certainly too narrow for such a large building. It is surely a mistake that the windows of the wards should be divided into small panes by wooden frames. I can only conclude that this has been done for the sake of architectural effect; but such windows are unsuitable, because the frames only make unnecessary ledges for collection of dust.

### Absence of Cross-ventilation.

Strangely enough, the service rooms are provided with plate-glass windows. The lavatories open directly upon the corridors without any cross-ventilation, and this arrangement is common to other modern hospitals that I have visited, both in Canada and the United States; whereas in Great Britain and Europe it is considered absolutely necessary that all lavatories should be placed in sanitary towers which are separated from the main buildings.

### Absence of Light.

It is, I think, a very serious defect that several of the corridors and passages leading to the wards, etc., have been so constructed that they are entirely dependent upon artificial lighting for their illumination even upon the brightest days.

Taken altogether, however, this hospital may be regarded as the latest development in Canada of a modern institution for the treatment of the sick; and, I think, well deserves the high eulogium which has been pronounced upon it by Sir William Osler, who recently paid it a long visit and expressed his opinion, "That it was about as perfect as such an institution could be." Certainly its erection and equipment in the short space of just over two years reflects great credit upon the managers, architects, and builders.

